# Hyaluronan Inhibits Tlr-4-Dependent RANKL Expression in Human Rheumatoid Arthritis Synovial Fibroblasts

**DOI:** 10.1371/journal.pone.0153142

**Published:** 2016-04-07

**Authors:** Tatsuo Watanabe, Nobunori Takahashi, Shinya Hirabara, Naoki Ishiguro, Toshihisa Kojima

**Affiliations:** Department of Orthopaedic Surgery, Nagoya University Graduate School of Medicine, Nagoya, Japan; University of Leicester, UNITED KINGDOM

## Abstract

The Toll-like receptor (TLR) signaling pathway is activated in synovial fibroblast cells in patients with rheumatoid arthritis (RA). The receptor activator of nuclear factor-κB (RANK) and its ligand, RANKL, are key molecules involved in the differentiation of osteoclasts and joint destruction in RA. Hyaluronan (HA) is a major extracellular component and an important immune regulator. In this study, we show that lipopolysaccharide (LPS) stimulation significantly increases RANKL expression via a TLR-4 signaling pathway. We also demonstrate that HA suppresses LPS-induced RANKL expression, which is dependent on CD44, but not intercellular adhesion molecule-1 (ICAM-1). Our study provides evidence for HA-mediated suppression of TLR-4-dependent RANKL expression. This could present an alternative target for the treatment of destructed joint bones and cartilages in RA.

## Introduction

Rheumatoid arthritis (RA) is an autoimmune disease characterized by chronic inflammation and subsequent destruction of bone and cartilage of joints [1]. Structural damage can result in long-term disability. With the advent of synthetic disease-modifying antirheumatic drugs (DMARDs), such as methotrexate, and biological DMARDs, clinical remission and absence of inflammation and immunologic activity have become realistic goals in RA. However, biological DMARDs cannot completely suppress structural damage [2–4]. Clinical remission has also become a realistic goal in RA, although much remains unknown about the pathogenesis of RA. Several key molecules, signal mediators, and pathways are implicated in the pathogenesis of RA, one of which is the Toll-like receptor (TLR) [5].

Recent studies have implicated TLR signaling in the activation and direction of the adaptive immune system through upregulation of costimulatory molecules of antigen presenting cells [6]. TLRs belong to the family of pattern-recognition receptors and play a crucial role in the activation of the innate immune system in response to invading microorganisms [7]. In the joints of RA patients, exogenous and endogenous TLR ligands have been identified [8, 9], and in synovial fibroblast cells, TLR3 and TLR4 are highly expressed [10].

Osteoclasts are acid phosphatase-positive multinucleated cells that originate from hematopoietic stem cells, and are essential for skeletal remodeling and regeneration [11]. However, excessive osteoclasts often contribute to bone diseases such as arthritis, osteoporosis, and cancer bone metastasis. Focal bone loss in RA is mediated by several cells such as osteoclasts, activated CD4+ T-cells, synovial fibroblasts, stromal-osteoblasts, and synovial macrophages. Osteoclasts located at the pannus-bone interface and in subchondral locations are the principal cells responsible for focal bone loss in RA. Chondroclasts and osteoclasts are often found in erosive areas of RA joints [12].

The receptor activator of nuclear factor-κB (RANK) pathway regulates osteoclast differentiation and function [13]. RANK ligand (RANKL), a member of the tumor necrosis factor (TNF) family of cytokines, is a key molecule involved in the differentiation of osteoclasts in the presence of macrophage colony-stimulating factor. In inflammatory arthritis, the RANK/RANKL pathway is activated, resulting in deregulated bone remodeling. Several reports have shown that RANKL is expressed at sites of bone erosion in RA synovial membranes [14, 15]. Thus, RANKL may serve as a target for preventing joint destruction in RA synovial membranes.

Hyaluronan (HA) is a component of synovial fluid and cartilage matrix, with its central role being joint lubrication. Recent studies have demonstrated a role for HA as an immune regulator [16]. For instance, HA suppresses expression of matrix metalloproteinases, such as MMP-1 and MMP-3, in RA synovial fibroblasts (RSF). Saito et al. also demonstrated that an intra-articular HA injection improves RA symptoms [17]. However, little is known about its mechanism of action.

In this study, we investigated the regulation of RANKL expression by TLR-4 activation in RSF cells, and explored the suppressive effect of HA on RANKL expression. Our findings provide a new alternative target for the treatment of destructed joint bones and cartilages in patients with RA.

## Materials and Methods

### 1 Patients

Five RA patients fulfilling the 1987 revised criteria of the American College of Rheumatology [18] participated in this study. Patients were recruited from January 2010 to November 2012 at Nagoya University Hospital. Written informed consent was obtained from all patients. The experimental protocol was approved by the Ethics Committee of the Nagoya University Graduate School of Medicine (Approval number 73–2).

### 2 Isolation of RSF cells

RSF cells were isolated by enzymatic digestion of synovial tissue obtained from RA patients undergoing total knee arthroplasty. Tissues were minced into 4–5 mm pieces and treated for 12 h with 4 mg/ml type I collagenase (Worthington Biochemical, USA) in Dulbecco’s modified Eagle’s medium (DMEM) at 37°C in 5% CO_2_. Dissociated cells were centrifuged at 500 x g, re-suspended in DMEM supplemented with 10% fetal bovine serum (FBS), 100 units/ml penicillin, 100 μg/ml streptomycin, and 0.25 μg/ml amphotericin, and plated in 75 cm^2^ flasks.

### 3 Reagents

Lipopolysaccharide (LPS) was purchased from SIGMA-ALDRICH (Missouri, USA), and high molecular weight hyaluronan (HMW-HA) from KAKEN (Tokyo, Japan). The following antibodies were used: anti-TLR4 (HTA125)(Abcam, UK), anti-CD44 (BU52)(Ancell, Minnesota, USA), and anti-intercellular adhesion molecule-1 (ICAM-1) (84H10) (Beckman Coulter, Marseille, France).

### 4 Cell Culture

RSF cells were cultured in DMEM supplemented with 10% FBS at 37°C in a 5% CO_2_ humidified atmosphere. The medium was replaced every 3 days. When cells approached confluence, they were passaged at 1:4 with fresh medium. RSF cells from passages 4–7 were used in each experiment. For treatments, cells were serum starved overnight and stimulated with LPS (1 μg/ml) in the absence or presence of HMW-HA for 12 hours. Cell lysate was then collected for analyses. One hour before LPS stimulation, cells were pre-incubated with HMW-HA, and then treated with LPS and anti-TLR4, anti-CD44, or anti-ICAM-1 antibody. Anti-TLR4 antibody (Abcam, UK) was used to confirm that LPS stimulation signaled via the TLR4 pathway. Anti-CD44 (Ancell, USA) and anti-ICAM-1 (Immunotech, France) antibodies were used to investigate the potential HA receptor involved in the inhibitory effect of HMW-HA on LPS-induced RANKL expression.

### 5 Real-time RT-PCR

Total RNA was extracted using the RNeasy Mini Kit (Qiagen, Germany) according to the manufacturer’s instructions. Reverse transcription was performed with the High Capacity cDNA Reverse Transcription Kit (Applied Biosystems, USA) at 37°C for 120 min. Real-time RT-PCR was carried out using the Light Cycler System with FastStart Master SYBR Green PLUS (Roche, USA). The following primers were used: RANKL, forward primer 5’-ACCAGCATCAAAATCCCAAG-3’, reverse primer 5’-CCCCAAAGTATGTTGCATCC^3’; GAPDH, forward primer 5’-TGAACGGGAAGCTCACTGG-3’, reverse primer 5’-TCCACCACCCTGT-3’. All primers were obtained from Sigma-Aldrich Japan (Tokyo, Japan). PCR conditions were as follows: 2 min at 50°C and 10 min at 95°C, followed by 40 cycles of 15 s at 95°C and 1 min at 59°C. Data were collected during the last 30 s. Real-time PCR efficiencies and fold increase in mRNA copy number were calculated as previously described [19].

### 6 Western blotting

RANKL protein expression was evaluated by Western blotting analysis. Total protein was extracted using Cell Lysis Buffer (Cell Signaling, USA), which contained a protease inhibitor cocktail (Thermo, USA). Twenty micrograms of total protein per sample were loaded and separated by 10% SDS-PAGE under reducing conditions. Samples were transferred onto a nitrocellulose membrane and blocked in 5% nonfat dry milk. The membranes were then incubated with primary antibodies followed by incubation with horseradish peroxidase-conjugated secondary antibodies. Primary antibodies for RANKL (EPR4999) (Abcam, UK) and beta-actin (13E5)(Cell Signaling, USA) were used for analysis. Proteins were detected by chemiluminescence (Thermo, USA).

### 7 Statistical analysis

Values are expressed as mean ± standard deviation (SD). The Mann-Whitney U test was used for two-group comparisons, and the Kruskal-Wallis H test was used for multiple-group comparisons. The significance of individual differences was evaluated using the Mann-Whitney U test only if the Kruskal-Wallis H test indicated significance. P<0.05 was considered statistically significant. All statistical analyses were conducted with SPSS for Windows version 20 (IBM, Chicago, IL).

## Results

### 1 Induction of RANKL expression by TLR-4 activation

**Fig 1:**
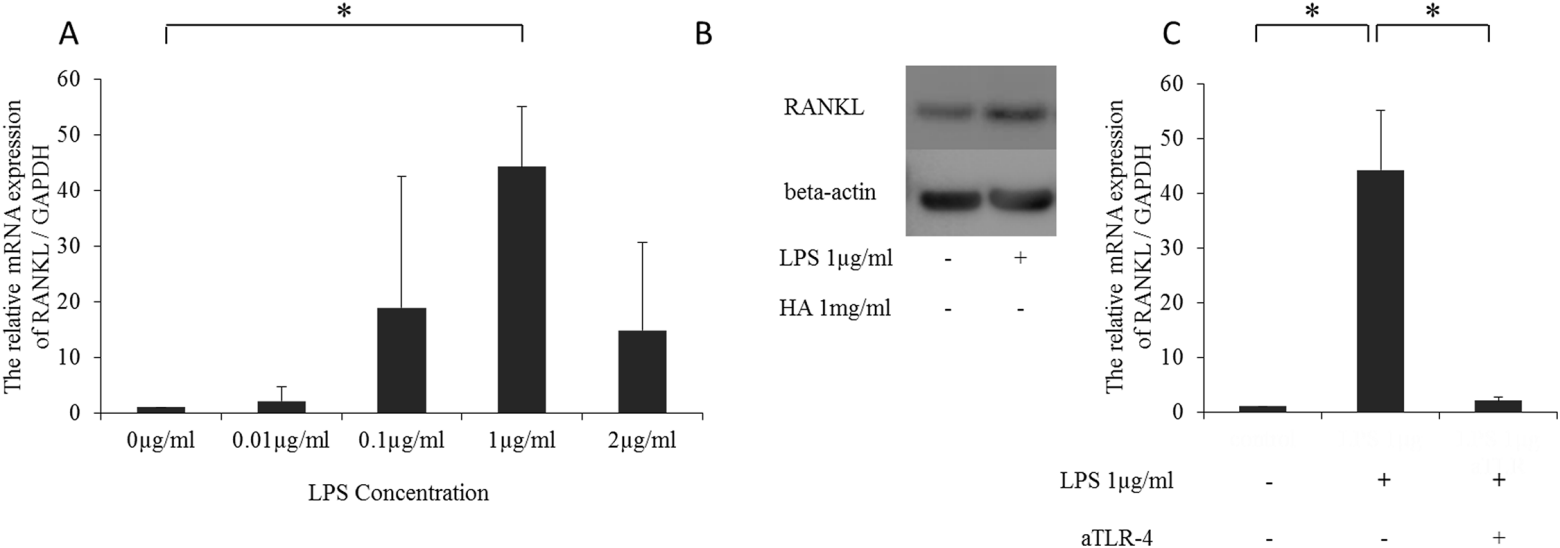
Expression of RANKL enhanced by LPS via TLR-4. (A) RANKL mRNA expression is induced by LPS. n = 5 independent replicates using 5 different samples. *p<0.05. P values were calculated by comparisons with the indicated sample. (B) Western blot analysis showing RANKL expression in human rheumatoid arthritis synovial fibroblasts. (C) Effect of anti-TLR4 monoclonal antibody pre-treatment on LPS-induced RANKL protein expression. n = 5 independent replicates using 5 different samples. *p<0.05.

We first confirmed whether LPS stimulation upregulates RANKL expression in RSF cells. As shown in Fig 1A, stimulation of RSF cells with LPS for 12 h significantly increased RANKL mRNA expression in a dose-dependent manner. The highest increase in RANKL mRNA expression was observed with 1μg/ml LPS. Western blotting analysis also showed an increase in RANKL protein expression (Fig 1B, S1 and S2 Figs). To demonstrate that the upregulation of RANKL expression was mediated by TLR-4, cells were pre-treated with an anti-TLR-4 antibody. As shown in Fig 1C, pre-treatment with the anti-TLR-4 antibody for 1 h clearly suppressed LPS-induced RANKL expression (Fig 1C).

### 2 Inhibition of TLR-4-dependent RANKL expression by HMW-HA

**Fig 2:**
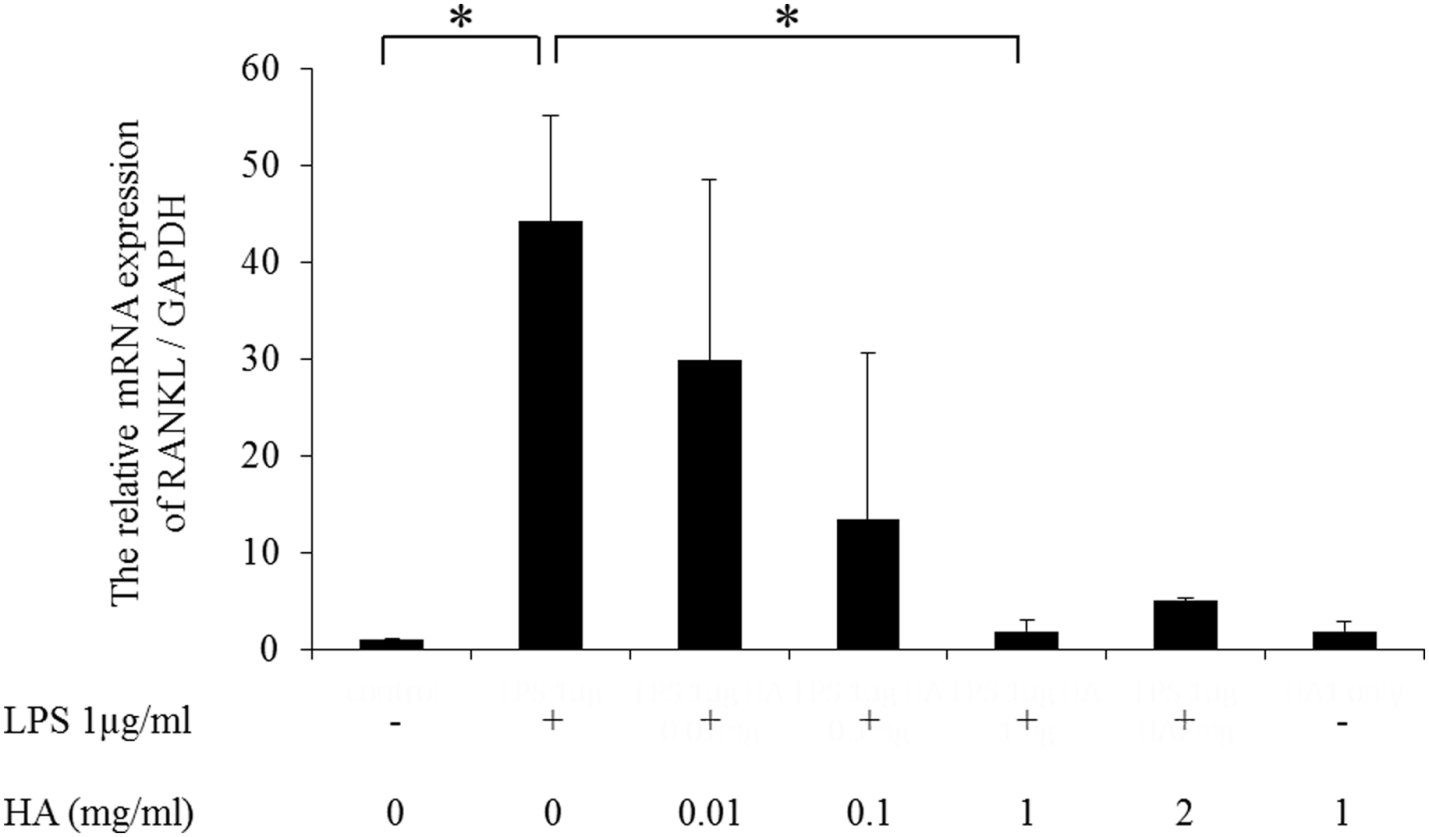
Inhibitory effect of HMW-HA on RANKL expression induced by LPS. Pre-incubation of cells for 1 hour and co-incubation with 1 mg/ml HMW-HA suppresses LPS-induced RANKL mRNA expression. This inhibitory effect was dose-dependent. HMW-HA alone had no effect. n = 5 independent replicates using 5 different samples. *p<0.05.

Pre- and co-incubation of RSF cells with HMW-HA clearly suppressed LPS-induced RANKL expression in a dose-dependent manner (Fig 2). LPS-induced RANKL mRNA expression was almost completely suppressed in the presence of 1 mg/ml HMW-HA. There was no significant effect on RANKL mRNA expression treated with HMW-HA alone (Fig 2).

**Fig 3:**
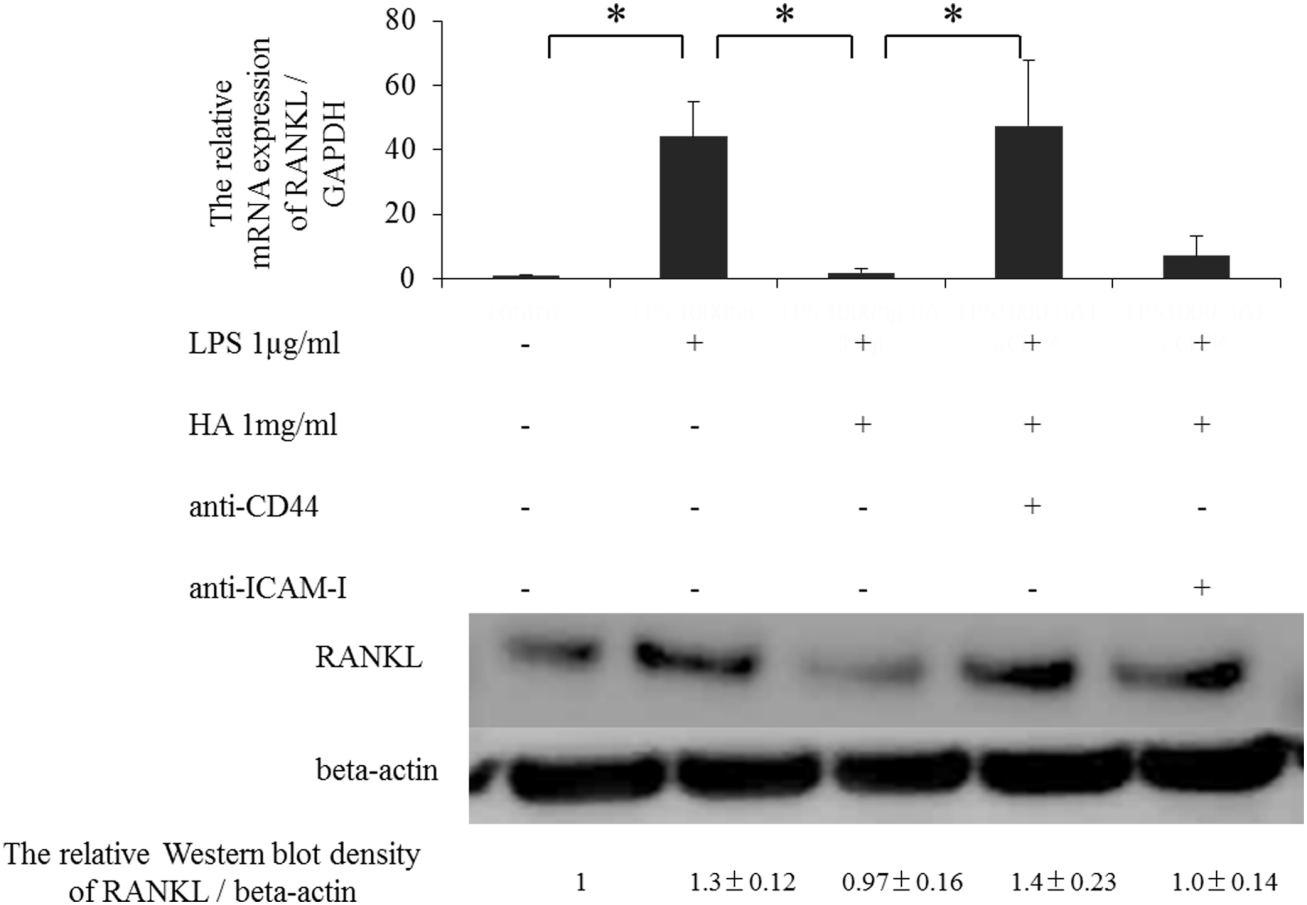
Inhibitory effect of HMW-HA via CD44 and ICAM-I on RANKL expression induced by LPS. Effect of HA treatment on LPS-induced RANKL mRNA expression. Messenger RNA expression data show the effects of pre-treatment with anti-CD44 and anti-ICAM-1 monoclonal antibodies on the inhibitory effect of HA on LPS-induced RANKL expression. n = 5 independent replicates using 5 different samples. *p<0.05 Western blot analysis shows RANKL expression in human rheumatoid synovial fibroblast cells treated with or without LPS, HA, anti-CD44 antibody, and anti-ICAM-1 antibody. n = 5 independent replicates using 5 different samples.

We next determined potential HA receptors involved in the increase in RANKL mRNA expression by pre-treating cells with an anti-CD44 or anti-ICAM-1 antibody for 1 hour. Pre-treatment with the anti-CD44 antibody reversed the suppression of LPS-induced RANKL mRNA expression by HMW-HA. In contrast, pre-treatment with the anti-ICAM-1 antibody had no significant effect (Fig 3, S3–S12 Figs). In addition, Western blotting analysis showed similar results as the mRNA expression data, i.e., LPS stimulated RANKL expression, which could be suppressed by HMW-HA. While pre-treatment with the ant-CD44 antibody suppressed the inhibitory effect of HMW-HA, pre-treatment with the anti-ICAM-1 antibody had no significant effect. These results suggest that CD44, but not ICAM-1, is the primary HA receptor involved in the inhibitory effect of HMW-HA on LPS-induced RANKL expression.

## Discussion

In this study, we demonstrated that HMW-HA suppresses TLR-4-dependent RANKL expression. We also found that this inhibitory effect was dependent on CD44, but not ICAM-1.

RANKL is a key molecule involved in bone and joint destruction in RA [20]. A targeted RANKL inhibitor, denosumab, reportedly suppresses structural damage in RA [21]. Since the absence of inflammation and immunologic activity can be achieved in clinical settings, suppressing RANKL expression and subsequent structural damage could be the next phase of RA treatment.

TLR-4 pathway activation is implicated both in the early [10] and late stages of RA pathogenesis [22]. In human RSF cells, TLR-4 is a component of one of the RANKL activating pathways for which LPS is the ligand [23]. Although we used a different concentration of LPS in our experiments, we also found that the increase of RANKL expression in human RSF cells was TLR-4-dependent, with an increase of about 40-fold in RANKL mRNA expression. However, only about a 30% increase was observed in RANKL protein expression, suggesting an involvement of negative signals after translation. As the present study did not investigate downstream signaling, a future study will be needed to further examine the lack of correlation between RANKL mRNA expression and protein expression.

CD44 and ICAM-1 are the main cell surface molecules associated with HA. Either CD44 or ICAM-1 is required for the anti-catabolic effects mediated by HA [24, 25]. In this study, pre-treatment of cells with anti-CD44 antibody, but not anti-ICAM-1 antibody, significantly reduced the inhibitory effect of HA on TLR-4-dependent RANKL expression. In RSF cells, CD44 and its downstream signaling elements, but not ICAM-1, play an important role in the inhibitory effect of HA on RANKL expression. In our recent study, we used a human fetal lung fibroblast cell line as a model for synovial fibroblasts, and demonstrated that HMW-HA has an inhibitory effect on cathepsin K in human fibroblasts [26]. Another study using mouse bone marrow stromal cells found that HA-CD44 interactions down-regulate RANKL expression via activation of the Rho kinase pathway [27]. These results suggest that HA-CD44 interactions play an important role in immune regulation.

Several HMW-HA preparations have been used to treat RA via intra-articular administration, and their effects on pain relief have been reported [28]. HA is a biodegradable, biocompatible, non-toxic, non-immunogenic, and non-inflammatory linear polysaccharide, making it an attractive drug. HA is well known for its utility in space filling and lubrication of joints, and the main reason for intra-articular administration of HA is for lubrication. However, the benefits of HA, such as pain relief, cannot be explained solely by its lubrication function. Recent studies have shown that HA is an immune regulator, and the present study found that HA suppresses TLR-4 dependent RANKL expression. The levels of HA in synovial fluid are significantly lower in RA patients than in healthy individuals [29]. Previous studies, as well as our present findings, show suppression of matrix metalloproteinases and RANKL by HA in RSF in clinical settings [30]. Restoration of HA to normal levels in the synovial fluid of RA patients may induce a protective effect against joint destruction by decreasing RANKL expression.

In this study, we confirmed that RANKL expression can be induced in a TLR-4-dependent manner, and suppressed by HMW-HA. Our findings provide a new alternative target for the treatment of destructed joint bones and cartilages in patients with RA.

## Supporting Information

S1 Fig Western blot analysis showing beta-actin expression in human rheumatoid arthritis synovial fibroblasts. This is beta- actin data, from left lane; control, LPS 1μg/ml.
(TIF)

S2 Fig Western blot analysis showing RANKL expression in human rheumatoid arthritis synovial fibroblasts. This is RANKL data, from left lane; control, LPS 1μg/ml.
(TIF)

S3 Fig Western blot analysis showing beta-actin expression in sample1 human rheumatoid arthritis synovial fibroblasts. This is beta- actin data, from left lane; control, LPS 1μg/ml, LPS 1μg/ml + HA 1mg/ml, LPS 1μg/ml + HA 1mg/ml + anti-CD44, LPS 1μg/ml + HA 1mg/ml + anti-ICAM-I.
(TIF)

S4 Fig Western blot analysis showing RANKL expression in sample1 human rheumatoid arthritis synovial fibroblasts. This is RANKL data, from left lane; control, LPS 1μg/ml, LPS 1μg/ml + HA 1mg/ml, LPS 1μg/ml + HA 1mg/ml + anti-CD44, LPS 1μg/ml + HA 1mg/ml + anti-ICAM-I.
(TIF)

S5 Fig Western blot analysis showing beta-actin expression in sample2 human rheumatoid arthritis synovial fibroblasts. This is beta- actin data, from left lane; control, LPS 1μg/ml, LPS 1μg/ml + HA 1mg/ml, LPS 1μg/ml + HA 1mg/ml + anti-CD44, LPS 1μg/ml + HA 1mg/ml + anti-ICAM-I.
(TIF)

S6 Fig Western blot analysis showing RANKL expression in sample2 human rheumatoid arthritis synovial fibroblasts. This is RANKL data, from left lane; control, LPS 1μg/ml, LPS 1μg/ml + HA 1mg/ml, LPS 1μg/ml + HA 1mg/ml + anti-CD44, LPS 1μg/ml + HA 1mg/ml + anti-ICAM-I.
(TIF)

S7 Fig Western blot analysis showing beta-actin expression in sample3 human rheumatoid arthritis synovial fibroblasts. This is beta- actin data, from left lane; control, LPS 1μg/ml, LPS 1μg/ml + HA 1mg/ml, LPS 1μg/ml + HA 1mg/ml + anti-CD44, LPS 1μg/ml + HA 1mg/ml + anti-ICAM-I.
(TIF)

S8 Fig Western blot analysis showing RANKL expression in sample3 human rheumatoid arthritis synovial fibroblasts. This is RANKL data, from left lane; control, LPS 1μg/ml, LPS 1μg/ml + HA 1mg/ml, LPS 1μg/ml + HA 1mg/ml + anti-CD44, LPS 1μg/ml + HA 1mg/ml + anti-ICAM-I.
(TIF)

S9 Fig Western blot analysis showing beta-actin expression in sample4 human rheumatoid arthritis synovial fibroblasts. This is beta- actin data, from left lane; control, LPS 1μg/ml, LPS 1μg/ml + HA 1mg/ml, LPS 1μg/ml + HA 1mg/ml + anti-CD44, LPS 1μg/ml + HA 1mg/ml + anti-ICAM-I.
(TIF)

S10 Fig Western blot analysis showing RANKL expression in sample4 human rheumatoid arthritis synovial fibroblasts. This is RANKL data, from left lane; control, LPS 1μg/ml, LPS 1μg/ml + HA 1mg/ml, LPS 1μg/ml + HA 1mg/ml + anti-CD44, LPS 1μg/ml + HA 1mg/ml + anti-ICAM-I.
(TIF)

S11 Fig Western blot analysis showing beta-actin expression in sample5 human rheumatoid arthritis synovial fibroblasts. This is beta- actin data, from left lane; control, LPS 1μg/ml, LPS 1μg/ml + HA 1mg/ml, LPS 1μg/ml + HA 1mg/ml + anti-CD44, LPS 1μg/ml + HA 1mg/ml + anti-ICAM-I.
(TIF)

S12 Fig Western blot analysis showing RANKL expression in sample5 human rheumatoid arthritis synovial fibroblasts. This is RANKL data, from left lane; control, LPS 1μg/ml, LPS 1μg/ml + HA 1mg/ml, LPS 1μg/ml + HA 1mg/ml + anti-CD44, LPS 1μg/ml + HA 1mg/ml + anti-ICAM-I.
(TIF)
