# Supplementary figures and images for: Hyaluronan Inhibits Tlr-4-Dependent RANKL Expression in Human Rheumatoid Arthritis Synovial Fibroblasts
Source: PLoS One. 2016 Apr 7;11(4):e0153142. doi: 10.1371/journal.pone.0153142 (PMC4824426; doi:10.1371/journal.pone.0153142)

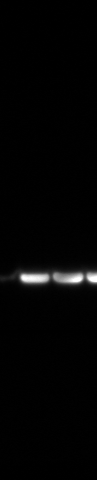

Supplement: S1 Fig — This is beta- actin data, from left lane; control, LPS 1μg/ml. (TIF) [file pone.0153142.s001.tif]

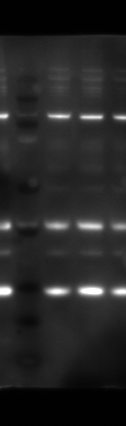

Supplement: S2 Fig — This is RANKL data, from left lane; control, LPS 1μg/ml. (TIF) [file pone.0153142.s002.tif]

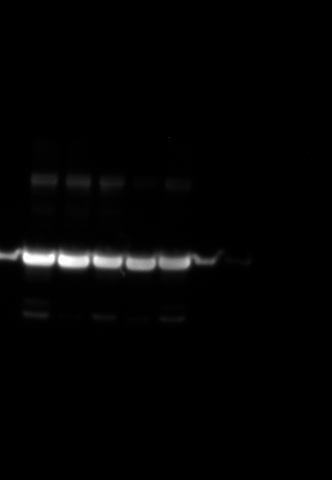

Supplement: S3 Fig — This is beta- actin data, from left lane; control, LPS 1μg/ml, LPS 1μg/ml + HA 1mg/ml, LPS 1μg/ml + HA 1mg/ml + anti-CD44, LPS 1μg/ml + HA 1mg/ml + anti-ICAM-I. (TIF) [file pone.0153142.s003.tif]

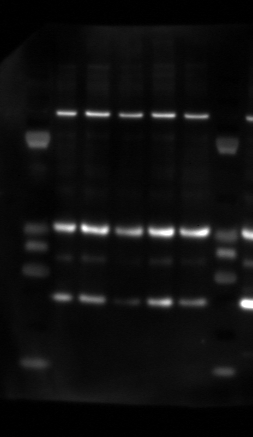

Supplement: S4 Fig — This is RANKL data, from left lane; control, LPS 1μg/ml, LPS 1μg/ml + HA 1mg/ml, LPS 1μg/ml + HA 1mg/ml + anti-CD44, LPS 1μg/ml + HA 1mg/ml + anti-ICAM-I. (TIF) [file pone.0153142.s004.tif]

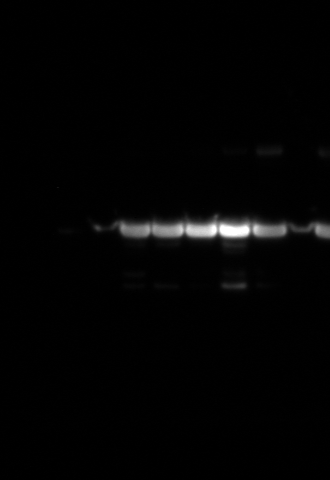

Supplement: S5 Fig — This is beta- actin data, from left lane; control, LPS 1μg/ml, LPS 1μg/ml + HA 1mg/ml, LPS 1μg/ml + HA 1mg/ml + anti-CD44, LPS 1μg/ml + HA 1mg/ml + anti-ICAM-I. (TIF) [file pone.0153142.s005.tif]

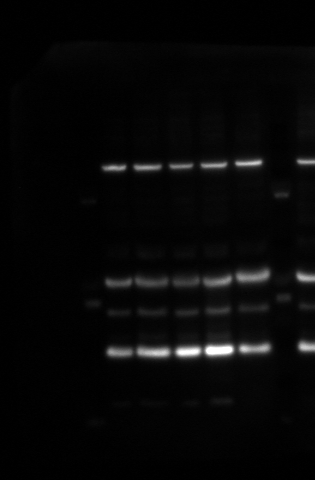

Supplement: S6 Fig — This is RANKL data, from left lane; control, LPS 1μg/ml, LPS 1μg/ml + HA 1mg/ml, LPS 1μg/ml + HA 1mg/ml + anti-CD44, LPS 1μg/ml + HA 1mg/ml + anti-ICAM-I. (TIF) [file pone.0153142.s006.tif]

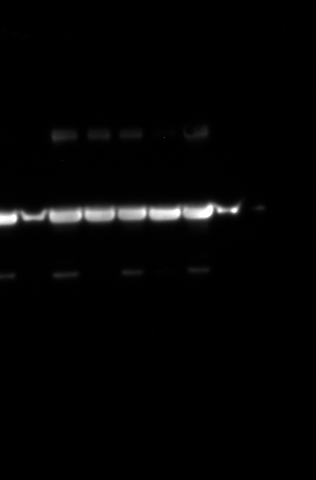

Supplement: S7 Fig — This is beta- actin data, from left lane; control, LPS 1μg/ml, LPS 1μg/ml + HA 1mg/ml, LPS 1μg/ml + HA 1mg/ml + anti-CD44, LPS 1μg/ml + HA 1mg/ml + anti-ICAM-I. (TIF) [file pone.0153142.s007.tif]

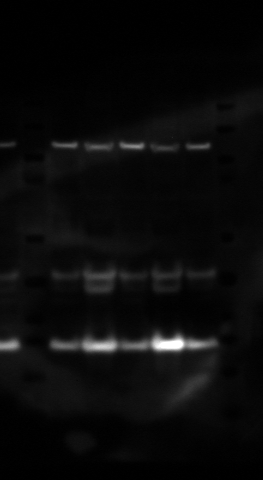

Supplement: S8 Fig — This is RANKL data, from left lane; control, LPS 1μg/ml, LPS 1μg/ml + HA 1mg/ml, LPS 1μg/ml + HA 1mg/ml + anti-CD44, LPS 1μg/ml + HA 1mg/ml + anti-ICAM-I. (TIF) [file pone.0153142.s008.tif]

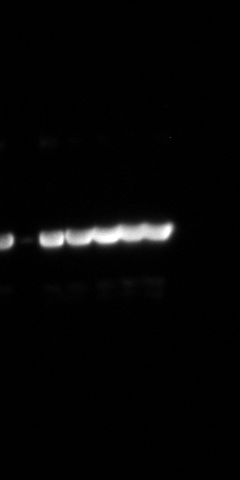

Supplement: S9 Fig — This is beta- actin data, from left lane; control, LPS 1μg/ml, LPS 1μg/ml + HA 1mg/ml, LPS 1μg/ml + HA 1mg/ml + anti-CD44, LPS 1μg/ml + HA 1mg/ml + anti-ICAM-I. (TIF) [file pone.0153142.s009.tif]

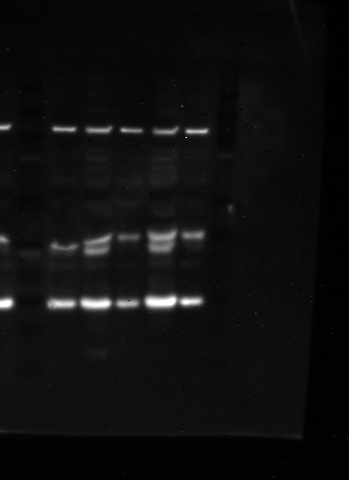

Supplement: S10 Fig — This is RANKL data, from left lane; control, LPS 1μg/ml, LPS 1μg/ml + HA 1mg/ml, LPS 1μg/ml + HA 1mg/ml + anti-CD44, LPS 1μg/ml + HA 1mg/ml + anti-ICAM-I. (TIF) [file pone.0153142.s010.tif]

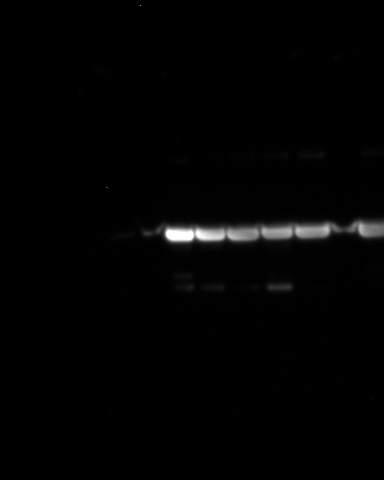

Supplement: S11 Fig — This is beta- actin data, from left lane; control, LPS 1μg/ml, LPS 1μg/ml + HA 1mg/ml, LPS 1μg/ml + HA 1mg/ml + anti-CD44, LPS 1μg/ml + HA 1mg/ml + anti-ICAM-I. (TIF) [file pone.0153142.s011.tif]

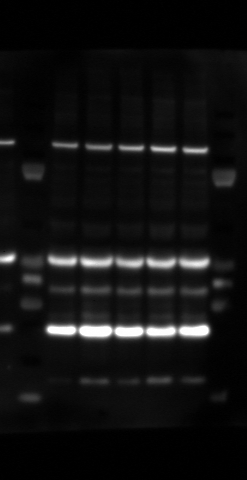

Supplement: S12 Fig — This is RANKL data, from left lane; control, LPS 1μg/ml, LPS 1μg/ml + HA 1mg/ml, LPS 1μg/ml + HA 1mg/ml + anti-CD44, LPS 1μg/ml + HA 1mg/ml + anti-ICAM-I. (TIF) [file pone.0153142.s012.tif]
